# The Late Dr. Lucius Clark

**Published:** 1879-01

**Authors:** H. P. Merriman

**Affiliations:** Chicago


ARTICLE XII.

CHICAGO, Dec. 12th, 1878.

*Editor Chicago Medical Journal and Examiner:*

So many of your readers have had a loving friendship for Dr. Lucius Clark, whose obituary appeared in your last number, that perhaps a few additional words of appreciation will find an acceptable place in your columns.

The writer has known him well for many years, and desires to express his thorough respect for Dr. Clark's character, both as a physician and a man. He was full of an enthusiastic love for his profession, which induced him to seize every moment he could spare from a laborious practice to study upon the subjects pertaining to his cases. His shelves were full of the best medical works, and the best journals of our country were always upon his table. He said to the writer, some twenty years ago, "Unless I am unusually fatigued, I make a point, before I retire, of looking over whatever journals come during the day, for I always get some good out of them." Not only did he read much, but he pondered upon it until it became thoroughly his own. Prof. Armor, of Long Island Medical College, wrote of him: "I have always regarded Dr. Clark as one of the most sagacious and able practitioners that I ever met at the bedside." Prof. Armor but expressed the general feeling in regard to him as was shown by the frequency with which he was sought in consultation by the physicians of Rockford and the neighboring towns.

He possessed a remarkable influence over young men. The writer remembers the keen interest and delight with which, when a boy at college, he and his classmates listened to the conversation of Dr. Clark, which not only interested and instructed, but always stimulated to higher attainment. Few possessed this power in a higher degree or used it better than he. He loved jokes, but sarcasm, ridicule and coarseness were beneath him. Wherever he went, he carried an atmosphere of good cheer and refinement.

So thorough was his love for his profession, that it affected young men like a contagion, making them think no work so noble or so grand as the physician's, and in consequence he was continually sought by those wishing to study medicine. It was his invariable custom to urge upon these the need of a liberal education first, saying, "the people continually require better educated men for their physicians, and if you would succeed, you should take a collegiate course or its equivalent before commencing professional studies."

Students from his office have nearly every year matriculated in the medical schools of Chicago or New York. His two sons have chosen their father's profession, and are working earnestly in it.

Dr. Clark was a religious man, taking great pains to be regular in his attendance at church, and his pastor always felt that in him he had a true helper, but his piety was of a kind that showed itself in deeds rather than words.

His memory will be warmly cherished in many hearts.

H. P. MERRIMAN.

---

IN order to facilitate the introduction of the metric system, as well as to furnish a convenient posological table, The Chicago Metric Club have published a Visiting List Dose Book, in which the doses are given both in the metric and in the apothecaries' weights and measures. It is light, and neatly bound in flexible silk covers, so that it may be carried in the visiting list or pocket without inconvenience. All physicians interested are urged to aid in placing this little book in the hands of the profession, not only by sending for it themselves but by ordering it for their friends. Sent free on receipt of six cents in postage. Address, THE METRIC CLUB, 181 Clark Street, Chicago.

